# Insights into the mechanism of vascular endothelial cells on bone biology

**DOI:** 10.1042/BSR20203258

**Published:** 2021-01-19

**Authors:** Ying Yin, Qingming Tang, Mengru Xie, Li Hu, Lili Chen

**Affiliations:** 1 Department of Stomatology, Union Hospital, Tongji Medical College, Huazhong University of Science and Technology, Wuhan 430022, China; 2 School of Stomatology, Tongji Medical College, Huazhong University of Science and Technology, Wuhan 430022, China; 3 Hubei Province Key Laboratory of Oral and Maxillofacial Development and Regeneration, Wuhan 430022, China

**Keywords:** Bone biology, endothelial cells, Extracellular vesicles, Juxtacrine, paracrine signalling

## Abstract

In the skeletal system, blood vessels not only function as a conduit system for transporting gases, nutrients, metabolic waste, or cells but also provide multifunctional signal molecules regulating bone development, regeneration, and remodeling. Endothelial cells (ECs) in bone tissues, unlike in other organ tissues, are in direct contact with the pericytes of blood vessels, resulting in a closer connection with peripheral connective tissues. Close-contact ECs contribute to osteogenesis and osteoclastogenesis by secreting various cytokines in the paracrine or juxtacrine pathways. An increasing number of studies have revealed that extracellular vesicles (EVs) derived from ECs can directly regulate maturation process of osteoblasts and osteoclasts. The different pathways focus on targets at different distances, forming the basis of the intimate spatial and temporal link between bone tissue and blood vessels. Here, we provide a systematic review to elaborate on the function of ECs in bone biology and its underlying mechanisms based on three aspects: paracrine, EVs, and juxtacrine. This review proposes the possibility of a therapeutic strategy targeting blood vessels, as an adjuvant treatment for bone disorders.

## Introduction

Both bone resorption and bone formation are important aspects of having strong and well-functioning bones throughout life [1]. Among many direct and indirect factors influencing osteogenesis and bone resorption, blood vessels in bone tissue are a notable and significant factor for coordinating these two activities. In addition to taking on the role of transportation and substance exchange, blood vessels also participate in various processes of bone development, reconstruction, and repair. During both endochondral and intramembranous ossification, blood vessels are the pioneers, leading to subsequent osteogenesis [2,3]. In other words, angiogenesis is one of the essential elements in bone defect healing [4,5].

The interaction between cells can be divided into three types: paracrine, extracellular vesicles (EVs), and juxtacrine. Paracrine activity involves the secretion of proteins/peptides and hormones, which are usually diluted during diffusion [6]. The different concentrations of paracrine factors affect different responses in target cells. EVs comprise exosomes, microvesicles, and apoptosomes containing growth factors, proteins, bioactive lipids, mRNA/miRNA, and DNA, and are perceived as mediators for intercellular communication even across distant tissues [7]. Juxtacrine interaction relies on the interaction between transmembrane ligands on one cell and receptors on the cell membrane of a neighboring cell. In bone tissue, most of the blood vessels are capillaries that make direct contact with pericytes, without the basement membrane between them; this makes the interaction between endothelial cells (ECs) and their pericytes tighter [8]. As a result, ECs in bone tissue have abundant opportunities to communicate with surrounding cells through juxtacrine, paracrine, and EVs. This review is based on these three aspects and describes the effects of microvascular ECs on surrounding cells in bone tissue, which finally affect osteogenic metabolism and bone resorption. We believe that clarifying the molecular mechanisms that underlie ECs participating in bone biology will not only further update our recognition of the pathogenesis of bone disorders but also afford new potential targets for treating bone diseases.

## The function of vascular ECs in osteogenesis and osteoclastogenesis

Bone formation and resorption are complicated processes. Hence, it is not straightforward to elucidate the function of ECs at the stages of bone development, remodeling, and regeneration. Both osteogenesis and osteoclastogenesis are closely coupled with angiogenesis. However, in the past, the coupling between angiogenesis and osteogenesis has attracted more attention. Numerous studies have evaluated the various functions and regulations of blood vessels in osteogenesis, which provide an opportunity to understand their role in bone biology more comprehensively. During endochondral bone formation, new blood vessels grow and transport osteoclast and osteoblast progenitors into the center of the future bone, as a first step in the process of bone formation [2]. Although intramembranous osteogenesis is poorly understood compared with endochondral bone formation, current studies conclude that small-bore capillaries invade into the initial ossification site at the initial stage of intramembranous osteogenesis and endochondral bone formation [3]. Furthermore, osteodistraction models have also shown that angiogenesis predominantly occurs before osteogenesis [9]. Blood vessels can lead to longitudinal growth of long bone by regulating cartilage resorption [10]. Numerous studies have shown the importance of angiogenesis in the process of bone, which is accompanied by the various signaling factors in the hematoma or blood clot [4,5,11]. Additionally, confocal microscopy revealed that mature osteoblasts gather around blood vessels that invade into the cartilaginous callus tissues during fracture healing [2]. Furthermore, evidence indicated the presence of signaling factor cross-talk from ECs to osteoclast lineage cells to promote migration from the circulatory system to bone tissue and osteoclastic differentiation. After recognition by ECs, monocytes can pass through endothelial gaps into bone tissue.

Based on recent reports, bone microvessels are divided into three subtypes, including type H, type L, and type E [8,12]. Based on the protein level of CD31 and endomucin (Emcn) in ECs, capillary vessels in bone tissue are defined as type H (CD31^high^ Emcn^high^) and type L (CD31^low^ Emcn^low^) blood vessels [8]. The former is known to play a vital role in bone development, inducing ossification. Another study proposed that type E blood vessels appearing in the embryonic and early postnatal bone, as the third EC subtype in bone tissue, supported osteoblast lineage cells more strongly than type H blood vessels and could transform into other EC subpopulations [12].

Additionally, another kind of cell—mesenchymal stem cells (MSCs)—that is indicated to be the same type of cell as pericytes [13] can pass through endothelial gaps. MSCs dwell in the perivascular niche of almost all mature tissues and will mobilize and migrate into damaged tissues to promote tissue healing [13–15]. Migration of MSCs from other tissues into bone is critical for bone repair [16].

In summary, blood vessels in bone tissue perform multiple functions, mostly because of EC-derived signaling molecules. This review elaborates the role of these molecules on bone biology including paracrine, juxtacrine, and secreted protein or other substances in EVs.

## EC-secreted cytokines play a vital role in bone biology

**Figure 1:**
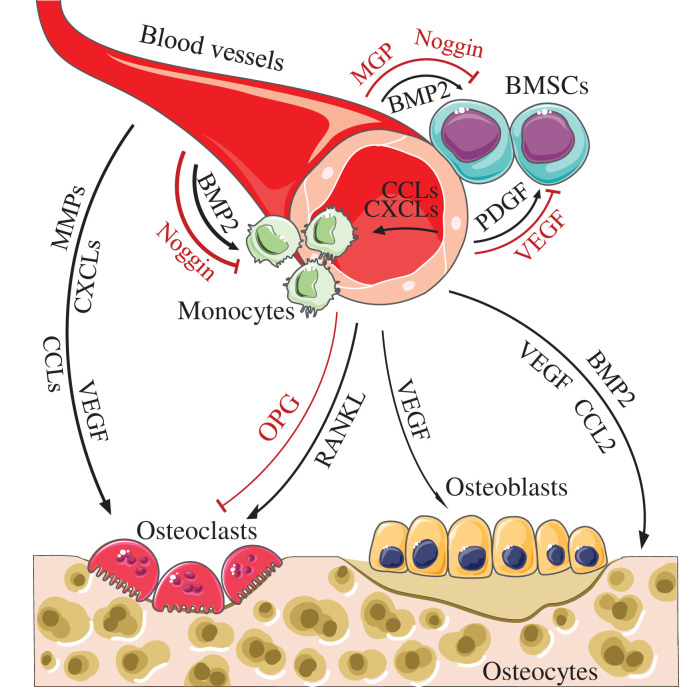
The effects of EC-secreted paracrine factors on osteogenesis and osteoclastogenesis In bone tissue, PDGF can recruit pericytes/MSCs, promote their growth, and inhibit their osteogenesis, and antagonize VEGF. BMP2 can promote osteogenesis, antagonize MGP, and Noggin. Besides, BMP2 can attract monocytes to adhere to ECs by antagonizing Noggin. VEGF can induce osteoclasts to migrate and affect osteoblastic differentiation. Furthermore, RANKL can induce osteoclastic differentiation by antagonizing OPG. As for inflammatory cytokines, CCLs and CXCLs can induce monocytes to migrate into bone tissue and differentiate into osteoclasts. MMPs can promote osteoclastic differentiation. Meanwhile, BMP2, VEGF, and CCL2 can prevent osteocyte apoptosis.

A number of previous studies have shown that ECs secrete several signaling molecules by paracrine interaction, such as platelet-derived growth factor (PDGF)-BB, vascular endothelial growth factor (VEGF), bone morphogenetic protein (BMP) 2, matrix Gla protein (MGP), receptor activator of nuclear factor-κB ligand (RANKL), and osteoprotegerin (OPG), which play important and critical roles in osteogenesis and bone resorption (Figure 1). The concentration of these cytokines are reduced as they move away from the blood vessels, resulting in limited effect. As reported by Francis and Palsson, the maximal distance a solitary cell *in vitro* can effectively communicate is approximately 250 μm by soluble cyto- and chemokines [17]. This distance can be further regulated if the cytokines bind to the extracellular matrix (ECM). Another study pointed out that this distance also depended on the strength of the enhanced-release time and rate [18]. Meanwhile, the degradation of released molecules would further limit the distance [19]. These factors determine the paracrine molecules secreted by ECs focus on the target cells close to blood vessels.

Several studies have shown a comprehensive perspective of PDGF in bone tissue. A recent study estimated that PDGF-BB in bone marrow was predominantly from TRAP^+^ cells (72.6%), while 12.6% were from ECs and 14.8% from other bone marrow cells [20]. Previous studies have shown that that PDGF-BB from macrophage-lineage TRAP^+^ cells could recruit Nestin^+^ and LepR^+^ periosteum-derived cells to the periosteal surface for periosteal bone formation [21]. Meanwhile, EC-derived PDGF-BB could recruit PDGFR-β-expressing pericyte progenitors into the new bone area [22,23]. The attached pericytes could stabilize the structure of newly formed blood vessels [24,25]. On the one hand, PDGF-BB can induce MSCs proliferation through PI3K signaling, while on the other, PDGF-BB can regulate the differentiation of MSCs via Erk signaling [26]. Another *in vitro* experiment also revealed that PDGF-BB can promote the proliferation of pericytes [27]. Besides, other studies also pointed out that PDGF-BB/PDGFR-β can increase the migratory response and proliferative capacity of MSCs but strongly inhibit osteogenic differentiation of MSCs [28–32]. After secretion from the ECs, PDGF binds to ECM via heparan sulfate proteoglycans and is confined to specific sites [33]. The interaction between PDGF and ECM promotes PDGF to retain itself close to ECs and form a chemoattracting gradient [34]. Simultaneously, it attenuates the effects of secretion rate and degradation of PDGF to the chemoattracting gradient. To sum up, the chemoattracting gradient of PDGF contributes to MSCs aggregation and benefits MSCs to maintain self-renewal and proliferation. When MSCs are far away from the blood vessels, the inhibition of osteogenesis from ECs-derived PDGF-BB will be weakened. Such a phenomenon may also occur in other secretory factors.

VEGF in bone tissue is mainly produced by hypertrophic chondrocytes, and some VEGF is secreted by newly formed ECs [35]. Quiescent ECs *in vitro* did not express VEGF [35]. However, stimulated by FGF2, quiescent ECs can be activated to form new capillaries and express both VEGF mRNA and protein. Additionally, hypoxia can also stimulate ECs to secrete VEGF [36], which occurs in fractured hematomas. Additionally, VEGF can inhibit the migration and proliferation of MSCs through PDGF receptors [37]. This is in line with a study wherein VEGF can antagonize PDGF-stimulated pericyte recruitment to regenerate blood vessels during angiogenesis [38]. In other words, in the progress of neovascularization, VEGF reduces vascular pericyte coverage and causes vessel destabilization [38]. Based on the retinal angiogenesis model, it was found that the signal across the angiogenic front was up-regulated with the loss of pericyte coverage [39]. This could likely be because reduction in vascular pericyte coverage caused by VEGF benefits blood vessels to sprout more easily during angiogenesis. Apart from strong regulation of angiogenesis, VEGF also plays an influential role in recruiting monocytes and osteoclasts, as well as regulating osteoclast differentiation [40–44]. VEGF can also regulate the fate of cartilage and inhibition of VEGF benefits cartilage fates [45,46], which play a crucial role in bone development when blood vessels invade the cartilage. In terms of osteogenesis, a previous study showed that VEGF could promote bone mesenchymal stromal cells to proliferate and show osteogenic differentiation [47]. This study showed that appropriate VEGF could promote osteogenesis, while a high dose of VEGF could inhibit osteogenesis [48]. The deletion of VEGF receptor 2 in osteoblastic lineage cells increased the maturation of osteoblast and mineralization in intramembranous ossification-mediated bone repair. However, an *in vitro* experiment showed contrary effects of VEGFR2 in that its activation promoted the survival of osteocytes [49]. Taken together, VEGF, as a paracrine factor, can work on a variety of cells and play a complicated role at an early stage of bone development. EC-derived VEGF can affect the pericytes surrounding ECs to a certain degree, especially during the period of angiogenesis. As for the effect of EC-derived VEGF on the whole bone tissue, a previous study shows that *Vegfa^fl/fl^ VE-cadherin-Cre* mice do not show significant differences in bone healing of a tibial monocortical defect model, which contrasted with findings from the littermate controls [48].

As a member of the TGF-β superfamily, BMPs can stimulate MSCs and osteogenic lineage cells to undergo osteoblast differentiation through the canonical BMP-SMAD pathway [50], which is vital in the earliest step of fracture healing [51]. The BMP-SMAD pathway can also prevent osteocytes, the terminal stage of osteogenic differentiation, from apoptosis [52]. Among the BMP family, BMP2 and BMP4 play a role in the interaction between ECs and pericytes. Under pathological stimuli such as the inflammatory microenvironment, the expression of BMP2 and BMP4 will have a strong response in ECs [53,54]. Nonetheless, a previous study showed that targeted deletion of *Bmp2* in vascular ECs did not impact fracture healing in any way [55]. Recent immunohistological studies of BMP2 expression showed that BMP2 was most strongly expressed in periosteal cells and hypertrophic chondrocytes [56]. BMP2 can also be released by pre-hypertrophic chondrocytes, osteoblasts, and osteocytes during the progression of endochondral healing [56,57]. Taken together, we can conclude that EC-derived BMP2 plays an insignificant role in fracture healing. With respect to bone development, EC-derived BMP2 has little effect on postnatal skeletal growth, structure, and strength [58]. BMP4 is a weaker stimulator of osteogenesis than BMP2 [59], and it is not required for limb skeletogenesis, bone formation, and fracture healing [60]. On the other hand, ECs can also secrete BMP antagonists such as MGP, follistatin, and Noggin through exocytosis. In healthy bone tissue, EC-derived MGP is supposed to interact with BMP2 to inhibit ossification [61], which regulates the differentiation of pericytes around ECs. Another article showed that Noggin was the main BMP antagonist secreted by ECs in bone tissue, regulating the differentiation of pericytes and thereby osteogenesis and promoting chondrocyte maturation [62]. If the balance of BMPs was broken, vascular calcification or tibial dyschondroplasia would occur [61,63]. Interestingly, BMP2 also plays a part in the adhesion of monocytes to ECs [64], ultimately affecting osteoclast formation. At the same time, Noggin or other BMP antagonist can interfere with monocyte migration by inhibition of BMP2 signaling [64], thereby decreasing the number of osteoclasts.

During osteoclast differentiation, RANKL and macrophage-colony stimulating factor (M-CSF) both played important roles. The latter could not be secreted by vascular ECs, rather only lymphatic ECs [65]. Patricia et al. first revealed that microvascular ECs can express both mRNAs of RANKL and OPG [66]. In a later study, it was shown that under pathological conditions, ECs stimulated by TGF-β could increase the expression of RANKL to promote osteoclastogenesis to benefit bone remodeling [67]. EC-derived RANKL plays a role in the differentiation of osteoclasts, which can be demonstrated by a phenomenon that the absence of EC-derived RANKL reduced the number of osteoclasts around ECs along with the total number of osteoclasts [10]. As for OPG which can bind to RANKL to block the interaction of the latter with RANK on the osteoclast membrane, it was noted that OPG could also be synthesized by vascular ECs [68]. Malyankar et al. found that at least some EC-derived OPG were associated with the surface of ECs such as a juxtacrine factor; normally, OPG does not contain any transmembrane domain [68]. However, this study did not prove whether the OPG binding to the surface of ECs still had the capacity to interact with RANKL. Another *in vitro* study showed that ECs from various tissues could secrete OPG to inhibit the differentiation of osteoclasts [69]. Lekesiz et al. revealed that, in ECs, the expression level of OPG was negatively correlated with RANKL [70]. Taken together, it may be concluded that vascular ECs normally inhibit osteoclast differentiation, which is consistent with a phenomenon that, in a healthy state, osteoclasts are typically found around the trabecular bone rather than blood vessels. Osteocytes strongly express OPG and are the major source of RANKL [71,72]. Besides, Kehmia et al. pointed out that OPG was mainly derived from B cells in the bone microenvironment [73]. Those two pieces of evidence further prove the limitation of RANKL and OPG deriving form ECs.

In pathological conditions, ECs are an important source of inflammatory cytokines. After undergoing ionizing radiation, ECs overexpressed CX3CL1 that attracted circulating CX3CR1^+^/CD11b^+^ cells and induced the latter to undergo osteoclast differentiation [74]. Additionally, CX3CL1 stimulated ECs to secrete other inflammatory chemokines like CXCL2 and CXCL12 in the form of autocrine signaling. A recent study showed that CXCL10, CCL2, and CCL5 have similar osteoclastogenic effects, with the latter especially possessing the largest chemotactic effect on osteoclast progenitors [75]. In another study, under hypoxic or ischemic conditions, cardiac microvascular ECs significantly increased the production of CXCL10 [76]. There is a study comparing the chemokine secretion ability of ECs from different vascular beds [77]. This study determined that CXCL8 and CCL2 could be constitutively produced by human saphenous vein endothelium, lung and dermal microvascular ECs, human umbilical vein ECs (HUVECs), and a bone marrow EC (BMEC) line. Besides, CCL5 and CXCL10 were secreted only after those cells were stimulated by tumor necrosis factor-α (TNF-α) or interferon-γ (IFN-γ). A related phenomenon showed that human BMECs under the stimulation of parathyroid hormone-related protein can secrete CCL2 to promote the differentiation of osteoclast *in vitro* [78]. Interestingly, Kitase et al. reported that CCL2, at a low dose, can prevent apoptosis of osteocytes [79]. Additionally, an immunostaining result demonstrated that HUVECs stimulated by lipopolysaccharide or atorvastatin could secrete CCL19 and CCL21 [80]. In this study, it was also proven that CCL19 and CCL21 could induce monocytes to adhere and migrate to HUVECs. Furthermore, another study demonstrated that CCL19 and CCL21 could promote osteoclast’s capacity of resorption and migration [81]. Taken together, it can be stated that ECs can secrete some of the chemokines in a healthy state and mostly in an inflammatory state, inducing monocytes to migrate into bone tissue and differentiate into osteoclasts.

MMPs are a type of collagenases capable of regulating the progression of the embryo and physiological remodeling tissue as well as disease development [82]. A recent study showed that ECs from bone tissue could produce more MMPs such as MMP9 and MMP14 than those produced from osteoclasts. In the present study, the absence of EC-derived MMP9 resulted in the reduction in growth plate size [10]. This supports ECs-derived MMP9 as contributing to the process of blood vessels invading and degrading cartilage tissue during bone development. Meanwhile, the degraded cartilage matrix released large amounts of VEGF, inducing angiogenesis. Hence, MMPs can recruit osteoclasts in bone development [83]. Engsig et al. demonstrated that MMP inhibitors could completely prevent TRAP^+^ cells’ migration. Subsequently, another study confirmed that the deficiency of gelatinase B/MMP9 in mice caused delayed osteoclast recruitment, which affected early bone development [44]. As for MMP14, which is also called membrane-type 1 MMP, it can maintain osteoblasts and osteocytes survival through activating TGF-β [84]. However, another study revealed that MMP14 increased soluble RANKL production, thereby stimulating osteoclast formation and bone resorption [85].

## EVs derived from ECs are indispensable in bone biology

**Figure 2:**
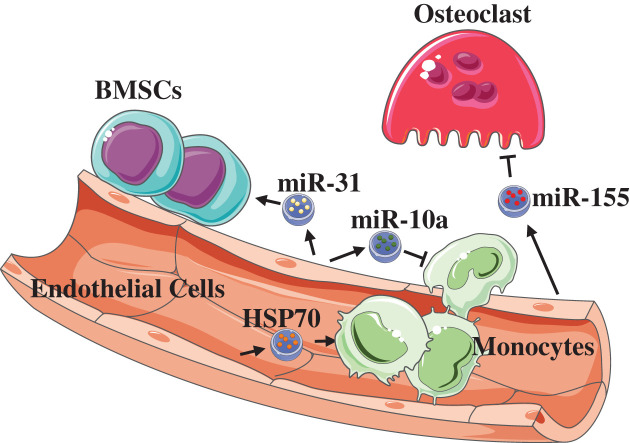
EC-derived EVs with different contents will have different effects in bone tissues EVs containing miR-31 can inhibit the osteogenesis of BMSCs. And EVs within HSP70 can promote the adhesion of monocytes. EVs within miR-10a will inhibit monocyte/macrophage activation. Besides, EVs containing miR-155 can suppress osteoclastic differentiation.

Besides size, EV populations can be categorized by additional qualifiers of identity-differential biogenesis, including exosomes (30–150 nm in diameter), microvesicles (50–1000 nm in diameter), and apoptosomes (50–5000 nm in diameter) [86]. Compared with the paracrine pathway, EVs can protect their contents such as sequestered proteins and mRNA from degradation and enable cell communication across tissues. Studies about EC-derived EVs are insufficient. The studies reporting the effect of ECs-derived EVs on bone tissue are even fewer. Alique et al. found that the number of total microvesicles secreted from senescent HUVECs was greater than that from young cells [87]. In senescent ECs, the secretion of EVs containing miR-31 is up-regulated [88]. Later, it was shown that miR-31 could regulate osteogenesis by targeting *Osx, Runx2*, and SATB2 [89,90]. Then, a recent study further reported that EC-derived EVs containing miR-31 could be taken up by bone MSCs (BMSCs), which inhibits the differentiation of osteogenesis through down-regulating the expression of FZD3, a Wnt5A receptor [91]. EVs contain a variety of substances. A previous study showed that under TNF-α stimulation, HUVECs could release more endothelial microparticles, one kind of EVs [92]; interestingly, the endothelial microparticles contained significant BMP2 which could promote osteogenic differentiation and was normally secreted by exocytosis [93]. The association between EC-derived EVs and osteoclasts was revealed gradually. Vítková et al. confirmed that EC-derived EVs can directly bind to monocytes, resulting in increasing transendothelial migration of monocytes [94]. Another study showed that EVs from quiescent ECs could regulate the inflammatory responses of monocytes; more importantly, these EVs inhibited monocyte/macrophage activation by transferring miR-10a into monocytic cells and targeting some components of the NF-κB pathway, such as *IRAK4* [95]. On the other hand, Zhan et al. showed that under the induction of oxidative low-density lipoprotein and homocysteine, ECs effectively increased the release of EVs which contained HSP70; and HSP70 could activate monocytes and induce them to adhere to ECs [96]. A recent study further confirmed the relation between ECs and osteoclasts through EVs [97]. This study showed that bone marrow-derived macrophages could internalize EC-exosomes that contained a large amount of miR-155, which then altered their morphology; and miR-155 could suppress osteoclastic differentiation. Additionally, it was found that EC-exosomes showed more effective bone targeting than exosomes derived from osteoblasts or BMSCs, which was confirmed with biophotonic imaging after injecting intravenous Dil-labeled exosomes. Another study revealed that ECs might direct the function of heterotypic cell types, systemically and specifically, via the contents contained within their EVs (Figure 2) [98].

## EC-contact regulation in bone biology is also an important method

Juxtacrine interaction is a cell–cell signaling mediated by the direct interaction between adjacent cells and plays a key role in cell growth, proliferation, and metabolism. Among various juxtacrine factors, the most studied signaling pathways are the ephrin-Eph and Notch. Some of them are involved in osteogenesis and osteoclastogenesis. As early as 1995, a juxtacrine model was reported in a study that bovine bone ECs had a certain adhesion to preosteoclastic cells by direct contact *in vitro* [99].

**Figure 3:**
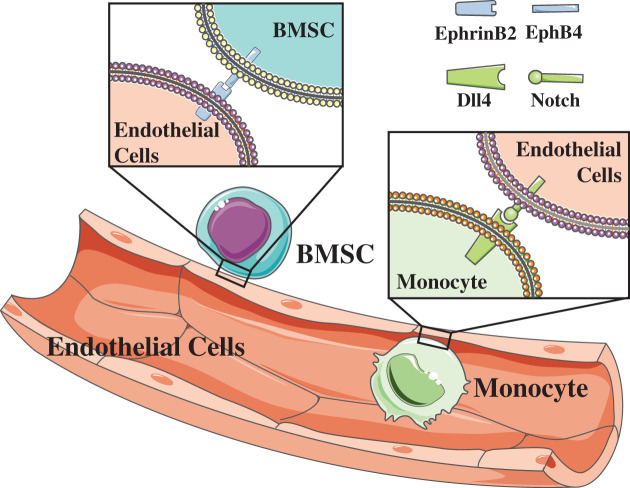
ECs interact with surrounding cells via juxtacrine factors on the membrane ECs-derived Dll4 can control the differentiation of monocyte/macrophage. In bone tissue, ECs promote HBMSCs to maintain self-renewal and proliferation by ephrinB2-EphB4 signaling.

Direct and indirect evidence from extensive studies showed the potential of pericytes to differentiate into osteoblasts, chondrocytes, adipocytes, smooth muscle cells, fibroblasts, and macrophages [100–105]. A recent study reported the role of direct interaction between ECs and pericytes in maintaining differentiation capacity [106]. This study showed that ECs promoted human BMSCs (HBMSCs) to maintain its self-renewal and stemness by the juxtacrine pathway—ephrin-Eph signaling (Figure 3). As signaling factors of bidirectional juxtacrine signaling, ephrins and Ephs can be ligands and receptors for each other, giving rise to bidirectional signal transduction and have been researched extensively. In the last century, studies have shown the interaction between ECs and surrounding mesenchymal cells through ephrin-Eph signaling [107]. In bone tissue, ephrins and Ephs are widely expressed in various cells (Table 1), and ephrinB2 is known to bind to EphB1-4, EphB6, and EphA4, while EphB4 can only bind to ephrinB2. Through the activation of bidirectional signaling pathways, the ephrin-Eph signaling pathway produces diverse effects in tissues. For example, the EphB4-ephrinB2 signaling pathway has been shown to repress osteoclast precursors from maturing through inhibiting c-Fos-NFATc1 cascade [108], which is consistent with another study that showed inhibition of ephrin-Eph signaling in osteoclastic differentiation [109]. On the contrary, ephrinB2 enhanced osteogenic differentiation through EphB4 of osteoblasts, which is a forward signaling [108,110]. Osteocytes with the absence of ephrinB2 increased more autophagosomes both *in vivo* and *in vitro* [111]. Besides, the stimulation of ephrinB2 affected the production of collagen type II in osteoarthritic chondrocytes, indicating its role in controlling chondrocyte differentiation [112]. Given all that, a reasonable conclusion may be reached in that arterial or venous ECs in bone tissue may interact with osteocytes, osteoblasts, and chondrocytes through ephrin-Eph signaling. However, relevant data are lacking.

**Table 1:** Cell distribution of components of ephrin-Eph signaling

Cell	Ligand	Receptor	References
EC	ephrinB2	EphB4	[106,107,159,160]
BMSC	ephrinB2		[106]
Osteoblast	ephrinB2	EphB2, -B4	[108,110]
Osteocyte	ephrinB2	EphB4	[111,161]
Osteoclast precursor and osteoclast	ephrinB2		[108,109]
Chondrocyte	ephrinB2	EphB4	[109]

As a vital parameter of the mammalian vascular system, the Notch pathway works by direct contact between two adjacent cells. In bone tissue, Notch ligands and receptors exist in various cells (Table 2). The Notch pathway can regulate MSC behavior as well as embryological bone formation [113–117]. Deleting Notch proteins in osteoclast precursors can enhance their osteoclastic differentiation and bone resorption [118]. As for mesenchymal progenitor cells, Notch2 can inhibit their differentiation that did not bias lineage allocation [115]. An *in vitro* experiment showed that inhibition of Notch signaling significantly decreased the expression of cyclin D1 in human MSCs, which indicated that the proliferation of human MSC was weakened [119]. A relevant conclusion was drawn in another research that the activation of Notch promoted the expansion of mesenchymal progenitor cells [120]. Further, this research revealed that Notch1 and -2 could control the fate of MSCs and their differentiation towards osteoblastic lineage cells, while Notch2 could suppress osteoblast differentiation [120]. However, another study showed Jagged1 could induce BMSCs to osteoblastic differentiation through Notch2 [121]. Thus, some studies support that Notch promotes osteogenesis [117,122,123], while others do not [118,120,124,125]. Besides, an *in vitro* experiment showed that mice with conditional deletion of *Rbpj* (a downstream molecule of Notch) in osteoblastic cells did not have an appreciable defect in osteoprogenitor cells [62]. These controversial results implied the complex role of the Notch pathway in regulating osteoblastic differentiation. A series of studies have shown that Jagged1 was up-regulated highly during the reparation of bone fracture and could induce HBMSCs to differentiate into osteoblasts [121,126,127]. In another study, while both Notch ligands, Jagged1 and Dll4, had opposite effects on angiogenesis in the skin [128], the latter could block endothelial activation and reduce angiogenesis through Notch1 signaling, and the former could block Dll4-Notch1 signaling to allow endothelial activation by VEGF and endothelial layer growth. Taken together, the complex role of the Notch pathway in bone tissue may be considered from the perspective of diverse Notch ligands with opposite effects. Notch1 has been shown to play a more important role than Notch2 and Notch4 in vascular angiogenesis [62,129–131]. The pleiotropy effects of Notch signaling was also noted in another phenomenon Notch1 activated by Dll4 from adjacent ECs promoted EC proliferation, and vessel growth in bone tissue and indirectly promoted osteogenesis through secretion of Noggin [62], which was contrary to the phenomenon discovered in other organs and tumors that Notch in ECs represses vascular angiogenesis [128,132,133]. Given the existence of an interaction between ECs and other cells, it is likely that EC-derived Notch ligands may act upon Notch proteins on the cell membrane around blood vessels in bone tissue. A previously conducted review suggested the possibility of this hypothesis [134]. Additionally, a study showed that EC-derived Dll1 can transform Ly6C^hi^ monocyte into Ly6C^lo^ monocyte by Notch2, both *in vivo* and *in vitro* [135], which indicated that ECs can regulate the fate of monocytes through the Notch pathway and also likely act on the process of osteoclastic differentiation through the Notch pathway. Furthermore, some studies showed that EC-derived Dll4 controlled the differentiation of monocyte/macrophage in the heart (Figure 3) [136,137].

**Table 2:** Cell distribution of components of Notch signaling

Cell	Ligand	Receptor	References
EC	Dll1, -4 and Jagged1	Notch1, -2, -4	[62,131,135,162]
Osteoclast precursor		Notch1, -2, -3	[118]
BMSC and osteoblastic lineage cell		Notch1, -2	[120]
Mesenchymal progenitor cell	Dll1, -4 and Jagged1 Notch1, -2	Notch1, -2	[115,120]
Chondrocyte		Notch2	[120]

There are other juxtacrine signaling pathways between ECs and the cells around the blood vessels, such as the semaphorin signaling system [138]. A previous *in vitro* study showed that HUVECs can express Plexin-B1, which is a membrane receptor and can bind with semaphorin 4D, a membrane protein similar to the αVβ3 integrin heterodimer [27,139]. Semaphorin 4D is found expressed in premature and mature osteoclasts [140]. Under the stimulation of semaphorin 4D, ECs can make PDGF-B, restrain stem cells from transforming into pericytes [27].

In addition to the above signal pathways, some researchers have revealed other forms of contact between ECs and their surrounding cells. An *in vitro* experiment showed that direct contact of HBMSCs with HUVECs increased the expression of type I collagen and alkaline phosphatase activity [141]. In this study, both HBMSCs and HUVECs could express connexin43, which constituted a gap junctional channel and benefited cell-to-cell communication, as confirmed by immunocytochemistry. Interestingly, confocal microscopy has revealed the mitochondria transferred from MSCs to rat lung microvascular ECs through juxtacrine interaction [142], consistent with previous reports [143,144].

## The function of ECs in bone disease

Osteoporosis has become a major public health issue with a high risk of fractures, especially for postmenopausal women. Histological results showed that primary osteoporosis is characterized by decreased numbers of sinuses and arterial capillaries in bone tissue [145]. The same phenomenon occurs in postmenopausal osteoporosis [146]. Later, in the research about age-related bone loss, it was found that type L capillaries did not change significantly, while the number of type H vessels decreased in old mice [8]. Bisphosphonate is a commonly used medicine to treat osteoporosis, which is considered to play a function in osteogenesis. Recently and interestingly, it was found that bisphosphonate could enhance blood flow and angiogenesis in old mice [147]. This study further revealed that treatment with bisphosphonate in aged mice activated the Notch pathway in ECs and increased type H capillaries. Bisphosphonate’s effect on osteoporosis is partly achieved by its action on blood vessels. Another study also showed that the administration of recombinant SLIT3 could be a vascular-targeted medicine to treat osteoporosis, which promoted angiogenesis of type H vessels [148].

ECM remodeling is an important process of bone fracture healing. A previous experiment has shown that bone healing was delayed in *Mmp9*^−/−^ mice [149]. MMP9 is mainly produced by type H blood vessels [10]. Xu et al. found recombinant SLIT3 can also play a function in fracture healing by expanding type H vessels [148]. Therefore, the mechanism may be that ECs secrete MMPs to degrade the ECM and promote callus mineralization.

Radiation osteomyelitis usually occurs after head and neck radiation therapy, as a common and serious complication [150]. In an irradiated murine model, ECs overexpressed CX3CL1, resulting in recruiting circulating osteoclast precursor cells to the irradiated bone, which induced osteoclastic differentiation and bone loss [74]. Subsequent experiments have confirmed that knockout of CX3CL1 or using its antagonist could prevent osteoclastogenesis and ameliorate bone resorption. This finding provides a theoretical basis for targeted vascular therapy of bone resorption, beyond just radiation osteomyelitis.

Lung, prostate, and breast cancer, as the most common human cancers, are prone to bone metastasis, in which damage to bones is not negligible [151,152]. During bone metastasis of prostate cancer, osteoblasts and BMECs stimulated by prostate-derived parathyroid hormone-related protein increase the secretion of CCL2 that could enhance osteoclastic activity and prostate cancer growth in bone [78,153]. Meanwhile, anti-CCL2 treatment *in vivo* effectively suppressed the process. It has been reported that ECs played a major role in the secretion of CCL2 during bone metastasis of prostate cancer [154]. Therefore, it is feasible to target the secretion of inflammatory factors from blood vessels for osteoclastic diseases and bone metastasis, which may also apply to other diseases causing systemic inflammation.

## Discussion and outlook

In the treatment of bone tissue diseases, just as with osteoporosis and bone fractures, most attention was paid to promoting osteogenesis in the past. However, during such treatment, the drug targeting blood vessels was not thoroughly studied and widely used in the past. In the process of drug ingestion, blood vessels are the first stop for drugs to enter bone tissue. On the other hand, in addition to transporting nutrients, blood vessels, as mentioned above, also have multiple other functions that can affect bone tissue. Therefore, we put forward that a potential strategy to treat bone disease is using drugs targeting blood vessels in bone tissue.

During the transportation of drugs *in vivo*, EV can become an ideal carrier, which is an effective communication medium between cells even from different organs, both locally and remotely [155]. EC-derived EVs can target bone tissue [97]. A similar phenomenon was also found wherein EVs derived from osteoclasts could also target bone tissue [156]. The mechanism behind is worth studying in particular and has significant applications in the field of targeted therapy, which improves the curative effects of drugs in bone tissue and reduces the side effects of the drugs in other organs and tissues. Compared with the EVs derived from other cells, research about EVs released from ECs in bone tissue still has broad prospects and the related mechanisms are not completely understood, which is worthy of further in-depth exploration.

Most information pertaining to the role of ECs in bone tissue comes from research focusing on the coupling of angiogenesis and osteogenesis. However, our description of the ECs’ role in osteoclastogenesis is inadequate and incomplete. Therapeutic strategies that target inflammatory factors secreted from blood vessels such as CCL2 and CX3CL1 in some animal models of bone disease have proven successful and effective. The potential mechanism of regulation of osteoclastogenesis by ECs remains to be explored and studied. Similar to this, there are likely to be mechanisms that have not yet been elucidated that address the process of MSCs and osteoclast progenitors in peripheral blood entering into bone tissue, especially the mechanism of juxtacrine pathways.

The role of ECs in bone biology is still insufficiently clarified. For instance, osteocytes comprising approximately 95% of total bone cells have shown the role of osteocytes in hematopoiesis, myeloid differentiation, and lymphopoiesis [157,158]. Nevertheless, the ECs’ effects on osteocytes need further study. Additionally, there is another direction worth studying that the stimulation of a specific flow pattern generated by endothelial buds is important for bone tissue which is sensitive to mechanical stimulation [147]. Besides, ECs are not the main source of various cytokines in the bone matrix, which limits the influence of ECs on bone tissue and is contrary to the coupling between angiogenesis and osteogenesis. Additionally, research about these three EC subtypes is not comprehensive enough. It would be very interesting to further study the interconversion of these three EC subtypes.

Many of the above experimental results were found in mice, which means that they may likely not exist in an identical manner in the human body. Despite some of these limitations, we have the opportunity to further understand the detailed mechanisms by which ECs affect bone tissue and thereby target ECs as a potential therapeutic strategy for treating numerous bone diseases.

## Data Availability

The data are available from the corresponding author upon reasonable request.

## Abbreviations

BMEC: bone marrow endothelial cell
BMP: bone morphogenetic protein
BMSC: bone mesenchymal stem cell
CCL: C-C motif chemokine ligand
CXCL: C-X-C motif chemokine ligand
EC: endothelial cell
ECM: extracellular matrix
Emcn: endomucin
EV: extracellular vesicle
FGF: fibroblast growth factor
HBMSC: human bone mesenchymal stem cell
HUVEC: human umbilical vein EC
MGP: matrix Gla protein
MSC: mesenchymal stem cell
OPG: osteoprotegerin
PDGF: platelet-derived growth factor
RANKL: receptor activator of nuclear factor-κB ligand
TNF-α: tumor necrosis factor-α
VEGF: vascular endothelial growth factor
